# Entropy-Based Uncertainty-Aware Exploratory Factor Analysis for Ordinal Data: Application to Tramway Cultural Tourism Evaluation

**DOI:** 10.3390/e28060607

**Published:** 2026-05-28

**Authors:** Jiaozi Pu, Yaxin Shi

**Affiliations:** School of Smart Culture and Tourism, Chengdu University of Information Technology, Chengdu 610103, China; syx1020@126.com

**Keywords:** fuzzy exploratory factor analysis (FE-EFA), ordinal data, uncertainty-aware representation, Shannon entropy, Jensen–Shannon divergence, Likert-scale analysis, perception-based evaluation, tramway tourism

## Abstract

Background: Perception-based evaluation using Likert-scale survey data is widely applied in tourism and transport research, yet conventional point-valued encoding imposes artificial precision and overlooks ambiguity between adjacent ordinal categories. This limitation is particularly relevant in experiential contexts, where subjective judgments often involve transitional evaluations. Methods: This study develops a parameterized fuzzy–entropy exploratory factor analysis (FE-EFA) framework for uncertainty-aware analysis of ordinal perception data. The approach transforms ordinal responses into parameterized fuzzy membership distributions governed by a single effective uncertainty ratio, constructs a correlation structure in the five-dimensional membership space, and incorporates Shannon entropy and Jensen–Shannon divergence to characterize distributional dispersion and representation differences. The framework is applied to survey data from Chengdu Tramway Line 2 (*N* = 1242; 32 indicators). Results: Under the Kaiser criterion (eigenvalues > 1), conventional EFA yields a seven-factor structure, whereas FE-EFA identifies an additional eighth factor located near the retention boundary. Under a unified factor specification, both approaches preserve a consistent high-level structure, while FE-EFA shows fewer cross-loadings and a more differentiated loading pattern in this empirical case under the adopted exploratory specification. From an information-theoretic perspective, FE-EFA produces higher entropy (average = 0.8688) and low Jensen–Shannon divergence (average = 0.0133), suggesting a limited redistribution of ordinal information without substantially altering the overall distributional structure. Entropy-adjusted weighting further reveals systematic shifts in indicator importance across key dimensions. Conclusions: The FE-EFA framework extends conventional Likert-scale analysis by introducing an uncertainty-aware representation layer prior to factor extraction. It preserves overall structural stability while suggesting a more differentiated organization of latent constructs and indicator-level representations in this empirical context. The proposed approach provides an exploratory representation-level extension for perception-based evaluation and decision support in tramway cultural tourism development and related contexts.

## 1. Introduction

Tourist mobility is closely linked to transport infrastructure, which shapes the developmental trajectories of destinations [1,2]. Among urban transport modes, tramway systems occupy a distinctive position in cultural tourism contexts. Unlike buses operating in mixed traffic or metro systems detached from the urban surface, tramways function at street level and maintain continuous visual and spatial interaction with their surroundings. The travel process thus becomes an experiential interface through which urban landscapes are perceived and interpreted, positioning perception as a critical analytical dimension rather than a secondary outcome in tramway-based tourism studies.

In empirical research, such perceptual dimensions are typically measured using structured survey instruments, most commonly Likert-type scales [3,4,5,6]. However, the methodological implications of treating ordinal Likert responses as interval-scale data remain insufficiently examined. From a measurement-theoretic perspective, Likert responses indicate relative ordering without guaranteeing equal intervals between categories [7]. Nevertheless, they are routinely assigned numerical values and analyzed as interval data, thereby implicitly assuming uniform distances between adjacent categories—an assumption that has been repeatedly questioned [8,9]. This issue is particularly salient in experiential evaluation contexts, where respondents frequently exhibit hesitation or ambiguity between adjacent response levels.

Although prior research acknowledges the tension between ordinal measurement and interval-based statistical treatment [10,11,12], practical applications often rely on point-valued encoding for analytical convenience [13]. In exploratory factor analysis (EFA), this practice compresses adjacent-category ambiguity into single-valued representations, potentially suppressing representation-level uncertainty at the preprocessing stage. At its core, this gives rise to a representation–analysis mismatch: ordinal responses containing boundary ambiguity are reduced to precise numerical inputs, while EFA operates on correlation structures that implicitly assume metric continuity and measurement precision. As a result, the factor structure is extracted from a representation that may be structurally misaligned with the ordinal and uncertain nature of perception data.

Existing methodological extensions provide partial solutions but remain limited in scope. Fuzzy exploratory factor analysis preserves uncertainty through membership-based representations [14], yet typically assumes that fuzziness is introduced at the data collection stage, thereby restricting applicability in studies relying on conventional ordinal survey designs. Meanwhile, fuzzy set theory provides a formal mechanism for representing partial membership [15], and Shannon entropy offers a quantitative measure of distributional dispersion [16]. These approaches collectively suggest that variability and ambiguity in ordinal responses may contain structurally meaningful information rather than being treated solely as measurement noise [17,18,19]. However, they have rarely been integrated into a unified factor-analytic framework that operates directly on standard Likert-scale data while preserving uncertainty at the representation stage.

Consequently, three structural limitations persist in existing approaches:

1. Adjacent-category ambiguity is not explicitly represented at the data level;
2. Informational uncertainty is not systematically incorporated into latent structural analysis;
3. Formal comparisons between alternative preprocessing strategies remain underdeveloped.

To address these limitations, this study develops a parameterized fuzzy–entropy exploratory factor analysis (FE-EFA) framework. The proposed approach introduces an uncertainty-aware preprocessing stage that transforms ordinal responses into fuzzy representations, constructs an uncertainty-sensitive correlation structure, and integrates entropy-based measurement with divergence-based comparison within a unified analytical procedure. Rather than replacing conventional EFA, the framework provides a complementary representation-level extension that preserves ordinal ambiguity while maintaining structural interpretability.

The transformation is governed by a parameterized mechanism that controls adjacent-category ambiguity and central concentration, whose combined effect is expressed through a normalized ratio representing the overall level of uncertainty. This design enables flexible uncertainty representation without modifying the original questionnaire format. By incorporating entropy into the analytical process, the framework further quantifies the informational dispersion of perception data and explicitly links uncertainty to indicator-level and structural characteristics.

The framework is applied to a dataset comprising 1242 respondents and 32 indicators in the context of tramway cultural tourism evaluation in Chengdu. By comparing point-valued and uncertainty-aware analytical routes within a unified empirical setting, the study examines how different encoding strategies influence factor structures and their associated informational properties.

This study makes three main contributions. First, it develops a parameterized FE-EFA framework that enables uncertainty-aware preprocessing of conventional ordinal survey data without requiring changes to questionnaire design. Second, it introduces an information-theoretic perspective into factor analysis by incorporating Shannon entropy and Jensen–Shannon divergence, allowing both perceptual dispersion and representation-induced information differences to be explicitly quantified. Third, through an empirical comparison, it shows that uncertainty-aware preprocessing preserves the global factor structure while suggesting refined indicator-level information profiles and more differentiated structural patterns in this empirical case.

Overall, the novelty of this study lies in introducing a post hoc uncertainty-aware representation layer that bridges standard Likert-scale measurement and fuzzy analytical frameworks within a unified and operational factor-analytic procedure, thereby addressing the representation–analysis mismatch inherent in ordinal perception data.

## 2. Theoretical Background and Research Design

### 2.1. Experiential Dimensions of Mobility-Based Tourism Evaluation

The relationship between transport infrastructure and tourism extends beyond the facilitation of spatial movement. Transport systems function not merely as conduits connecting origins and destinations, but as constitutive components of the tourist experience itself [1,2]. This perspective is particularly relevant for street-level tramway systems, which maintain continuous visual and spatial interaction with surrounding urban environments. Unlike underground metro systems or isolated vehicular travel, tramways enable what can be conceptualized as embedded mobility, in which the journey itself forms an integral part of the destination experience.

In cultural tourism contexts, this embeddedness has direct implications for evaluation design. Tramway corridors often traverse areas characterized by distributed cultural resources, including architectural heritage, public spaces, and symbolic landscapes. Consequently, key evaluation attributes—such as visual coordination, cultural identity, environmental comfort, and service accessibility—are inherently perceptual rather than purely physical. These attributes cannot be adequately captured through objective operational indicators alone, but instead require perception-based measurement frameworks.

This shift from objective performance metrics to experiential evaluation introduces a fundamental methodological challenge. Perception-based indicators are intrinsically subjective, linguistically expressed, and often imprecise, with evaluations frequently distributed across adjacent ordinal categories rather than concentrated at a single level. As a result, the analytical treatment of such data must account not only for latent structural relationships but also for the uncertainty embedded in human judgment, thereby motivating the need for uncertainty-aware representation and analysis.

### 2.2. Representational Uncertainty in Ordinal Survey Responses

Perception data in tourism research are predominantly collected using Likert-type scales, which provide ordered categorical responses to capture subjective evaluations [3,4,5,6]. From a measurement-theoretic perspective, such responses are ordinal: they convey ranking information without ensuring equal distances between adjacent categories [7].

However, in empirical practice, these ordinal responses are routinely encoded as point-valued numerical variables and analyzed using statistical methods that assume interval-scale properties [8,13]. This practice introduces a representational simplification in which each response is treated as an exact numerical value, implicitly neglecting the ambiguity that may exist between adjacent categories.

This issue becomes particularly pronounced in experiential evaluation settings. When respondents assess attributes such as cultural atmosphere, visual quality, or symbolic meaning, their judgments often lie between adjacent categories rather than aligning precisely with a single discrete level. The assumption of adjacent-category ambiguity is supported by both psychometric theory and empirical studies on ordinal response behavior. Respondents map continuous internal evaluations onto discrete linguistic categories, a process that is inherently subject to threshold uncertainty [20,21]. Studies on fuzzy rating scales further suggest that respondents may express partial membership across adjacent categories, particularly in the middle range of the scale [9,22]. Under point-valued encoding, such boundary ambiguity is compressed into a single category choice, effectively discarding uncertainty at the data representation stage.

When these encoded values are subsequently used in exploratory factor analysis (EFA), the implications extend beyond measurement. EFA extracts latent structures based on correlation patterns among observed indicators [10,11,12]; if the input data have already undergone uncertainty compression, the resulting factor structure may reflect a representation shaped by point-valued simplification of the underlying perceptual landscape.

Existing approaches to this issue have primarily focused on modifying data collection instruments, for example through fuzzy Likert scales that allow respondents to express partial membership across categories [9]. While conceptually rigorous, such approaches require redesigning survey instruments and are therefore not readily applicable to widely used conventional datasets. This limitation motivates the need for a post hoc strategy that can recover uncertainty from standard ordinal responses without altering the original measurement design.

### 2.3. Parameterized Fuzzy Representation and Entropy Measurement

Fuzzy set theory provides a natural framework for representing partial membership and modeling uncertainty in categorical data [15]. Instead of assigning each observation to a single category, fuzzy representation allows a response to be distributed across adjacent categories with varying degrees of membership. When applied to ordinal survey data, this enables the explicit modeling of boundary ambiguity inherent in subjective judgments [9,15].

In this study, ordinal responses are transformed into fuzzy membership distributions through a parameterized mapping mechanism. The transformation is governed by two parameters with interpretable behavioral roles: boundary fuzziness ($\beta_{b}$), representing the degree of hesitation between adjacent categories, and central concentration ($\beta_{c}$), representing the respondent’s confidence in the selected category. Due to the normalization structure of the membership function, the effective shape of the distribution depends only on the ratio $r=\beta_{b}/\beta_{c}$, which serves as a single uncertainty parameter controlling the interpolation between crisp point-valued encoding (r = 0) and maximum admissible fuzziness (r approaching 1). The constraint r < 1 ensures that the observed category always retains the highest membership value, preserving ordinal primacy.

This parameterized construction is related to discrete triangular fuzzy numbers [23] and fuzzy linguistic representations [24], but is specifically adapted for bounded five-point ordinal scales with a boundary folding rule to handle endpoint categories. Unlike fuzzy Likert scales that require instrument-level modification [9], this approach is applied post hoc to standard ordinal data, enabling uncertainty-aware analysis of existing survey datasets. In practice, the ratio r is selected within a stable range identified through sensitivity analysis (Section 5.2), rather than optimized to a single value.

While fuzzy representation preserves uncertainty at the encoding stage, an additional mechanism is required to quantify its informational implications. Shannon entropy provides a measure of distributional dispersion, capturing the degree of uncertainty or spread within a probability distribution [16]. When applied to fuzzy response distributions, entropy reflects the extent to which evaluations are concentrated or dispersed across adjacent categories [17]. Furthermore, Jensen–Shannon divergence provides a symmetric and bounded measure for comparing point-valued and fuzzy distributions of the same indicator [25]. Together with entropy, it establishes an information-theoretic layer for evaluating representation-induced information differences.

### 2.4. Comparative Analytical Framework

The analytical strategy adopted in this study is explicitly comparative rather than substitutive. The proposed framework is not intended to replace conventional EFA, but rather to evaluate how uncertainty-aware representation influences factor-analytic outcomes under controlled conditions.

Two parallel analytical routes are constructed based on the same dataset and indicator system. The first follows the conventional approach, in which Likert responses are directly encoded as numerical values and used to construct a Pearson correlation matrix for factor extraction [10,11]. This serves as the baseline benchmark. The second route introduces an uncertainty-aware preprocessing stage. Ordinal responses are transformed into fuzzy membership distributions, preserving adjacent-category ambiguity at the data level [9,15]. These representations are then used to construct a correlation structure in membership space, enabling factor extraction under uncertainty-aware conditions. In parallel, entropy is computed to characterize distributional dispersion, and Jensen–Shannon divergence is used to quantify representation differences between the two encoding strategies [16,17,25].

By comparing these two analytical routes, the study examines whether incorporating uncertainty at the representation stage is associated with more differentiated structural patterns, interpretable indicator allocation, and sensitivity to representation-level variation. On this basis, the proposed fuzzy-entropy exploratory factor analysis (FE-EFA) is positioned as a representation-level extension of conventional EFA, rather than as a modification of its core estimation procedure.

## 3. Indicator System and Data Source

### 3.1. Indicator System

The indicator system employed in this study was developed through a two-stage procedure that integrated literature-based extraction and expert refinement. An initial pool of candidate indicators was constructed through systematic review of prior studies on tramway systems and cultural tourism evaluation. This pool was subsequently screened and consolidated through expert consultation to ensure conceptual relevance, clarity, and coverage of key experiential and operational dimensions.

The finalized questionnaire comprises 32 indicators covering various aspects of tramway-based cultural tourism evaluation. These indicators include, for example, track landscape, vehicle styling, passenger visual field, thermal environment, station spacing rationality, and tourism information services. Together, they capture both perceptual and functional characteristics of tramway systems and are consistent with the experiential evaluation perspective outlined in Section 2.

Empirical data were collected through a structured questionnaire survey conducted among users of Chengdu Tramway Line 2. Respondents were required to have direct experience with the tramway system to ensure the validity of perception-based evaluations. After data screening and validation, a total of 1242 valid responses were retained for analysis. All indicators were measured using a five-point Likert scale, generating ordinal response data suitable for both conventional and uncertainty-aware analytical treatments.

### 3.2. Data Collection and Sample Characteristics

The data used in this study were collected through a structured questionnaire survey conducted along Chengdu Tramway Line 2. To ensure adequate statistical power for exploratory factor analysis, the sample size was designed to satisfy the commonly recommended ratio of at least 5–10 observations per variable. Given 32 indicators, a minimum sample size of 160 respondents was required.

In total, 1300 questionnaires were distributed through two channels:

1. On-site distribution along the tramway corridor (900 copies).
2. Targeted distribution to university students from the University of Electronic Science and Technology of China and Southwest Jiaotong University, whose daily activity areas overlap with the tramway service region (400 copies).

A total of 1249 responses were returned, of which 1242 were valid after data screening, yielding an effective response rate of 95.54%. All respondents reported prior experience using the tramway system, ensuring the validity of perception-based evaluation.

Table 1 presents the demographic characteristics of the respondents. Among the valid respondents, 930 (74.88%) were local residents or long-term inhabitants of Chengdu, while 312 (25.12%) were non-local visitors. This composition reflects a mixed user structure, capturing both daily mobility users and tourism-related passengers.

### 3.3. Data Suitability Tests

To ensure the statistical validity of factor extraction and establish a baseline structure for comparison, standard diagnostic tests were conducted. As summarized in Table 2, the Kaiser–Meyer–Olkin (KMO) measure reaches 0.8755, indicating strong sampling adequacy, while Bartlett’s test of sphericity is highly significant ($\chi^{2}$ = 25,147.27, df = 496, *p* < 0.001), confirming that the correlation matrix is appropriate for latent structure detection. In addition, the Kaiser criterion (eigenvalues > 1) indicates a seven-factor solution for the conventional point-valued EFA, which serves as the baseline dimensionality for subsequent comparison.

This baseline structure provides a critical reference point for subsequent analysis. In the following section, the proposed fuzzy-entropy exploratory factor analysis (FE-EFA) framework is applied to the same dataset, enabling a controlled comparison between conventional point-valued representation and uncertainty-aware fuzzy representation in terms of factor structure, representation differences and interpretability.

## 4. Fuzzy-Entropy Exploratory Factor Analysis Framework

### 4.1. Parameterized Fuzzy Membership Representation

#### 4.1.1. Membership Construction

Let $X=(x_{ij}{)}_{n\times m}\;$ denote the original response matrix, where *n* is the number of respondents and *m* is the number of indicators. Each observation $x_{ij}\in \{1,2,3,4,5\}$ is an ordinal Likert-scale response.

Under conventional numerical encoding, each response is treated as a precise point value. However, from the perspective of fuzzy set theory [15], a categorical response can be more generally represented as a membership distribution over the set of admissible categories. This idea has been formalized through various constructions, including triangular fuzzy numbers [23], linguistic variables [24], and fuzzy rating scales for questionnaire data [22]. In this study, we adopt a discrete membership representation over the five ordered categories, which can be interpreted as a discrete analogue of a triangular fuzzy number with bounded support constrained to the ordinal scale. Unlike continuous fuzzy number representations, this formulation operates directly on the categorical support of the Likert scale and preserves the ordinal structure of the response space.

Specifically, each ordinal response is transformed into a fuzzy membership vector over the five response categories. As discussed in Section 2.2, the assumption that ordinal responses carry adjacent-category ambiguity is empirically grounded. The parameterized fuzzy construction formalized below provides a controlled mechanism for representing this ambiguity without requiring modifications to the original survey instrument.

(1) $$\mu_{ij}=(\mu_{ij1},\mu_{ij2},\mu_{ij3},\mu_{ij4},\mu_{ij5})$$

where $\mu_{ijr}\in [0,1]$ and

(2) $$\sum_{r=1}^{5}\mu_{ijr}=1$$

The membership values are constructed using two nonnegative parameters with explicit behavioral interpretations:

$\beta_{b}\geq 0$ (boundary fuzziness): Governs the degree of membership allocated to adjacent categories. A higher $\beta_{b}$ represents greater hesitation or ambiguity between the selected category and its neighbors, modeling the empirical tendency of respondents to perceive their evaluation as lying between two adjacent ordinal levels.

$\beta_{c}\geq 0$ (central concentration): Governs the degree of membership retained at the observed category. A higher $\beta_{c}$ represents greater confidence or decisiveness in the selected response.

Because all membership values are normalized by the factor $\left(2\beta_{b}+\beta_{c}\right)$, the resulting distribution depends only on the ratio $r=\beta_{b}/\beta_{c}$, which serves as a single effective uncertainty parameter. When r = 0, the fuzzy membership representation reduces to a crisp one-category membership representation, corresponding to the point-valued case at the representation stage; as r increases, membership is progressively redistributed toward adjacent categories. The constraint $\beta_{b}<\beta_{c}$ (equivalently, r<1) ensures that the observed category always retains the maximal membership value.

This single-ratio formulation improves interpretability while avoiding over-parameterization. Different uncertainty levels are therefore controlled through r, and sensitivity analysis is conducted across a range of parameter settings (Section 5.2).

The membership assignment rule is defined separately for interior and boundary categories. To simplify notation, define two derived coefficients:

(3) $$\alpha =\beta_{b}/(2\beta_{b}+\beta_{c})$$

(4) $$\gamma =\beta_{c}/(2\beta_{b}+\beta_{c})$$

These satisfy 2α+γ=1, so normalization is automatically guaranteed.

Interior categories ($x_{ij}=k\in \{2,3,4\}$): The membership of respondent i on indicator at category r is defined as the following piecewise function:

(5) $$\mu_{ijr}=\{\begin{array}{cc} \alpha , & r=k-1\\ \gamma , & r=k\\ \alpha , & r=k+1\\ 0, & otherwise \end{array}$$

The observed category receives the highest membership γ, while the two adjacent categories each receive α, forming a symmetric three-point distribution centered on k. This structure is analogous to a discrete triangular fuzzy number with unit-width support on each side.

#### 4.1.2. Boundary Folding and Representation Properties

Boundary categories ($x_{ij}=1$ or $x_{ij}=5$):

For boundary responses, the membership that would be assigned to a category outside the admissible range {1, …, 5} is folded back onto the boundary category.

When $x_{ij}=1$,

(6) $$\mu_{ijr}=\{\begin{array}{cc} \alpha +\gamma , & r=1\\ \alpha , & r=2\\ 0, & r\geq 3 \end{array}$$

When $x_{ij}$ = 5,

(7) $$\mu_{ijr}=\{\begin{array}{cc} 0, & r\leq 3\\ \alpha , & r=4\\ \alpha +\gamma , & r=5 \end{array}$$

This boundary folding rule ensures that all membership is allocated within the admissible support, normalization is preserved, and the boundary category absorbs the mass that cannot be distributed beyond the scale limits, resulting in asymmetric two-point endpoint distributions.

The constructed membership vector $\mu_{ij}$ satisfies the following properties for all valid parameter settings ($\beta_{c}>0$, ${{0\leq \beta }_{b}<\beta }_{c}$):

1. Non-negativity: $\mu_{ijr}\geq 0$ for all r.
2. Normalization: $\mu_{ij1}+\mu_{ij2}+\mu_{ij3}+\mu_{ij4}+\mu_{ij5}=1$.
3. Ordinal primacy: the observed category $x_{ij}$ receives the maximal membership value.
4. Locality: non-zero membership is confined to the observed category and its immediate neighbors.
5. Degeneracy: when $\beta_{b}=0$ (i.e., r = 0), the membership vector reduces to a unit vector $e_{k}$, recovering the conventional point-valued encoding.

Intuitively, the proposed transformation represents an ordinal response not as a single fixed category, but as a localized membership distribution centered on the observed response. The uncertainty ratio r controls the degree of redistribution toward adjacent categories. When r = 0, the framework reduces to conventional point-valued encoding.

#### 4.1.3. Numerical Illustration and Parameter Setting

Table 3 presents the complete membership vectors for all five possible responses under the selected parameterization $\beta_{b}$ = 0.20, $\beta_{c}$ = 0.60. This setting corresponds to an uncertainty ratio of $r=\beta_{b}/\beta_{c}\approx 0.333$ and is used in the main FE-EFA.

For comparison, under point-valued encoding (r=0), each individual response maps to a unit vector, such as category 3 being represented as (0, 0, 1, 0, 0). This representation has zero entropy at the individual-response membership level because all probability mass is concentrated in a single category. However, after aggregation across respondents, the indicator-level response distribution may still exhibit non-zero entropy, reflecting variation in observed responses across the sample. The selected parameterization introduces moderate dispersion: interior responses allocate 60% of the membership to the observed category and 20% to each adjacent category, forming a symmetric three-point distribution centered on the observed response. For boundary responses, 80% of the membership is assigned to the observed endpoint and 20% to the single adjacent category due to the boundary folding rule, resulting in an asymmetric two-point distribution. This parameterization preserves the dominance of the observed category while allowing limited redistribution to adjacent categories, thereby capturing moderate uncertainty without violating ordinal primacy.

The ratio *r* ≈ 0.333 was selected as a moderate uncertainty level within the empirically stable range identified through sensitivity analysis (Section 5.2). Under this setting, the observed category retains dominant membership while allowing limited redistribution toward adjacent categories. The main findings remain stable across the range r∈[0.10,0.50], within which the eighth eigenvalue consistently remains above the Kaiser threshold.

#### 4.1.4. Interpretation as Localized Ordinal Redistribution

The proposed membership construction can be interpreted as a localized ordinal redistribution process in ordinal space, where each response is replaced by a neighborhood-based membership distribution controlled by the uncertainty ratio r. Unlike standard kernel density estimation, the transformation operates at the individual-response level and preserves bounded ordinal support through the boundary folding mechanism. The present formulation intentionally adopts a minimal local-support structure to preserve interpretability and avoid excessive parameterization in ordinal uncertainty representation.

### 4.2. Membership-Space Covariance Modeling

#### 4.2.1. Construction of the Membership-Space Covariance Structure

Instead of collapsing fuzzy representations into scalar expected scores, the proposed framework models dependence directly in the fuzzy membership space. Let

(8) $${\bar{\mu }}_{j}=\frac{1}{n}\sum_{i=1}^{n}\mu_{ij}$$

denote the mean membership vector of indicator j.

The membership-space covariance between indicators j and k is defined as

(9) $$Cov_{f}(j,k)=\frac{1}{n}\sum_{i=1}^{n}(\mu_{ij}-{\bar{\mu }}_{j}{)}^{T}(\mu_{ik}-{\bar{\mu }}_{k})$$

The corresponding membership-space variance is

(10) $${Var}_{f}(j)={Cov}_{f}(j,j)$$

The normalized covariance measure is then given by

(11) $$\rho_{f}(j,k)=\frac{Cov_{f}(j,k)}{\sqrt{Var_{f}(j)Var_{f}(k)}}$$

The resulting normalized covariance matrix is given by

(12) $$R_{f}=(\rho_{f}(j,k){)}_{m\times m}$$

This formulation evaluates dependence directly in the fuzzy membership space through centered inner products of membership vectors, allowing ordinal uncertainty to be preserved throughout the representation process rather than collapsed into scalar-valued encoding. Consequently, adjacent-category ambiguity can enter factor analysis through the resulting covariance structure itself. The proposed framework may therefore be interpreted as covariance analysis in a simplex-constrained embedding space induced by fuzzy ordinal membership vectors.

#### 4.2.2. Relation to Ordinal Factor-Analytic Methods

Conventional exploratory factor analysis based on Pearson correlations has long been criticized for treating ordinal Likert-scale responses as interval-valued observations [7,8,26]. To address this issue, ordinal factor-analytic approaches such as polychoric correlation-based EFA have been widely adopted, where observed ordinal responses are assumed to arise from discretized latent continuous variables [26,27,28]. Compared with Pearson correlations, polychoric approaches are generally considered more appropriate for ordinal data under suitable distributional assumptions [26,28].

The proposed FE-EFA framework is not intended to replace polychoric or other ordinal factor-analytic methods. Instead, it focuses on a different methodological aspect, namely the representation of response uncertainty prior to latent structure extraction. Whereas polychoric approaches emphasize latent continuous-variable reconstruction through threshold estimation, FE-EFA introduces an uncertainty-aware representation layer that preserves adjacent-category ambiguity directly in the observed response space.

In this sense, the two approaches operate at different analytical levels and should be understood as complementary rather than substitutive. Systematic comparison between FE-EFA and alternative ordinal factor-analytic approaches, including polychoric EFA and item-response-based models, remains an important direction for future research.

### 4.3. Factor Extraction Under Uncertainty

Factor extraction is performed on the membership-space covariance matrix $R_{f}$. The eigenvalue decomposition of $R_{f}$ provides the spectral basis for determining the underlying latent structure.

Let $\lambda_{k}$ denote the *k*-th eigenvalue of $R_{f}$. The number of retained factors is determined using the Kaiser criterion, under which a factor is retained if

(13) $$\lambda_{k}>1$$

This criterion selects components that explain more variance than an individual standardized indicator. As the correlation structure $R_{f}$ is constructed in the fuzzy membership space, the resulting eigenvalues reflect the distributional characteristics of uncertainty-aware representations rather than point-valued approximations.

Accordingly, factor retention in the FE-EFA framework is directly governed by the spectral properties of the membership-space covariance matrix, allowing ordinal ambiguity to be incorporated into latent structure identification through the eigenvalue profile.

### 4.4. Entropy-Based Information Characterization

For each indicator j, let

(14) $$P_{j}=(p_{j1},p_{j2},p_{j3},p_{j4},p_{j5})$$

denote the empirical probability distribution of the original ordinal responses.

By averaging respondent-level membership vectors, the fuzzy category distribution of indicator j is obtained as

(15) $$Q_{j}=(q_{j1},q_{j2},q_{j3},q_{j4},q_{j5})=\frac{1}{n}\sum_{i=1}^{n}\mu_{ij}$$

Let $H_{j}$ denote the normalized Shannon entropy of indicator j. The normalized Shannon entropy of the fuzzy distribution is computed as

(16) $$H_{j}=-\frac{1}{\log5}\sum_{r=1}^{5}q_{jr}\log q_{jr}$$

where the convention 0log0=0 is adopted.

And the corresponding dispersion coefficient is

(17) $$D_{j}=1-H_{j}$$

This entropy-based characterization captures the degree of distributional dispersion induced by uncertainty-aware preprocessing. Higher entropy indicates greater ambiguity or response spread across adjacent categories, whereas lower entropy indicates a more concentrated fuzzy distribution.

### 4.5. Entropy-Structure Coupled Weighting

To incorporate both latent structural relevance and distributional concentration, the proposed framework constructs an entropy-structure coupled weighting scheme.

Let $\lambda_{k}$ denote the variance contribution of factor k, and let $l_{jk}$ denote the loading of indicator j on factor k. The variance-weighted loading contribution of indicator j is defined as:

(18) $$I_{j}=\sum_{k=1}^{K}\lambda_{k}l_{jk}^{2}$$

After normalization, the structural weight becomes:

(19) $$S_{j}=\frac{I_{j}}{\sum_{j=1}^{m}I_{j}}$$

The final entropy-adjusted weight is:

(20) $$w_{j}=\frac{S_{j}D_{j}}{\sum_{j=1}^{m}S_{j}D_{j}}$$

This construction assigns greater importance to indicators that are both structurally influential in the retained factor solution and relatively information-concentrated after fuzzy transformation.

### 4.6. Jensen–Shannon Divergence

To quantify the distributional difference between point-valued and fuzzy representations, the Jensen–Shannon (JS) divergence is employed [25]. For each indicator j, let $P_{j}=(p_{j1},p_{j2},p_{j3},p_{j4},p_{j5})$ denote the empirical probability distribution of the original ordinal responses, and let $Q_{j}=(q_{j1},q_{j2},q_{j3},q_{j4},q_{j5})$ denote the corresponding fuzzy distribution derived from membership aggregation.

The Jensen–Shannon divergence between $P_{j}$ and $Q_{j}$ is defined as:

(21) $$JS(P_{j}∥Q_{j})=\frac{1}{2}KL(P_{j}∥M_{j})+\frac{1}{2}KL(Q_{j}∥M_{j})$$

where the midpoint distribution $M_{j}$ is given by:

(22) $$M_{j}=\frac{1}{2}(P_{j}+Q_{j})$$

The Kullback–Leibler divergence is defined as:

(23) $$KL(P_{j}∥M_{j})=\sum_{r=1}^{5}p_{jr}\log\frac{p_{jr}}{m_{jr}},KL(Q_{j}∥M_{j})=\sum_{r=1}^{5}q_{jr}\log\frac{q_{jr}}{m_{jr}}$$

where $M_{j}=(m_{j1},m_{j2},m_{j3},m_{j4},m_{j5})$.

The average divergence across all indicators is computed as:

(24) $$JS_{avg}=\frac{1}{m}\sum_{j=1}^{m}JS(P_{j}∥Q_{j})$$

Since all indicator-level JS divergences are bounded within [0, 1], arithmetic averaging is adopted as a descriptive summary statistic. This divergence provides a symmetric and bounded measure of how uncertainty-aware fuzzy transformation redistributes category-level information relative to direct point-valued encoding, thereby serving as an indicator of representation shift introduced by the proposed framework.

### 4.7. Interpretation of the FE-EFA Framework

The proposed FE-EFA framework should be understood as a distribution-level extension of conventional EFA. Its main role is not to replace classical factor analysis, but to provide an uncertainty-aware analytical layer for ordinal perception data.

More specifically, the framework:

1. Preserves ordinal ambiguity at the representation stage through fuzzy membership construction;
2. Incorporates uncertainty directly into correlation modeling in membership space;
3. Enables factor extraction on an uncertainty-aware correlation structure;
4. Enhances interpretation through entropy measurement, divergence analysis, and entropy-adjusted weighting.

In this sense, FE-EFA extends the conventional point-valued workflow by introducing an exploratory uncertainty-aware representation layer before latent-structure analysis and by making the informational consequences of representation choices analytically visible.

### 4.8. Software and Implementation

All analyses were implemented in MATLAB R2024a. Conventional exploratory factor analysis (EFA) and the proposed fuzzy-entropy exploratory factor analysis (FE-EFA) were conducted under the same analytical conditions to ensure comparability.

For the conventional point-valued EFA, ordinal Likert responses were directly encoded as numerical values from 1 to 5. For FE-EFA, ordinal responses were transformed into fuzzy membership distributions using the parameterized uncertainty-aware mapping described in Section 4.1. The main analysis adopted an uncertainty ratio of r=0.333, corresponding to α=0.20 and β=0.60, which represents a moderate level of adjacent-category ambiguity.

The membership-space covariance matrix was constructed directly in the membership space following Equations (8)–(12). Factor extraction was based on eigenvalue decomposition of the corresponding correlation matrix, and factor retention was determined using the Kaiser criterion (eigenvalues > 1). To improve interpretability, Varimax orthogonal rotation was applied to the retained factor solution.

Cross-loadings greater than 0.30 were treated as substantial secondary loadings in factor interpretation. The threshold of 0.30 was adopted following common practice in exploratory factor analysis. Although this cutoff is widely used in the literature, the sensitivity of the factor structure to alternative cross-loading thresholds was not systematically examined in the present study. To partially assess robustness, sensitivity analysis was conducted across multiple uncertainty-ratio settings, evaluating the stability of factor retention and information-theoretic measures. Future research may further investigate the robustness of factor assignments under varying cross-loading criteria.

Shannon entropy and Jensen–Shannon divergence were computed using custom MATLAB scripts developed for this study. Supplementary robustness checks, including polychoric-correlation EFA, bootstrap resampling, split-sample replication, and simple-structure residual validation, were conducted as auxiliary diagnostics to assess the stability and ordinal-data robustness of the FE-EFA solution. The anonymized data and analysis code are available from the corresponding author upon reasonable request, subject to institutional data-sharing requirements.

## 5. Results and Discussion

### 5.1. Factor Retention and Factor Interpretation

#### 5.1.1. Factor Retention Under the Kaiser Criterion

To evaluate the effect of uncertainty-aware preprocessing on latent dimensionality, exploratory factor analysis was conducted for both approaches using the Kaiser criterion (eigenvalues > 1).

In addition to the Kaiser criterion, alternative factor-retention methods such as parallel analysis are often recommended for improved robustness. However, because the present study focuses on representation-induced differences between point-valued and uncertainty-aware analytical routes rather than on identifying an optimal factor-retention rule, the Kaiser criterion is adopted as a consistent exploratory benchmark for both methods. Applying the same retention rule ensures that observed differences in factor numbers can be attributed primarily to changes in the correlation structure introduced by uncertainty-aware representation.

It should be noted that the Kaiser criterion is used here as an exploratory retention rule and should therefore be interpreted with caution. Future studies may combine the proposed framework with parallel analysis, confirmatory factor analysis, or validation samples to further assess the stability and generalizability of the retained factor structure.

A sensitivity analysis was performed by varying the ratio between boundary fuzziness and central concentration. The results remain stable across a reasonable range, indicating that the factor retention outcome is not sensitive to parameter selection. A moderate uncertainty level was used in the main analysis.

**Figure 1:**
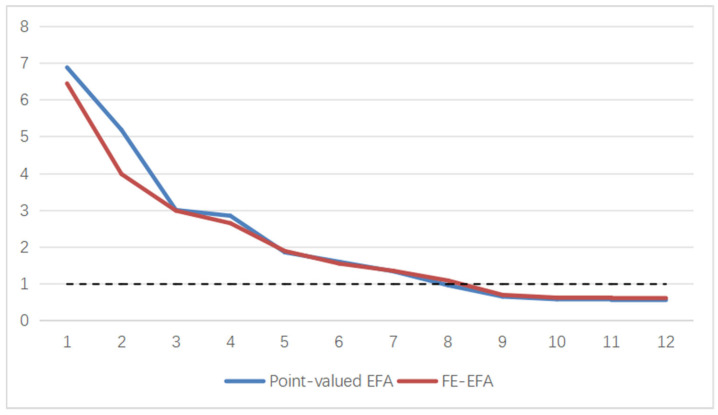
Scree plot with Kaiser cutoff.

As shown in Table 4, the point-valued EFA retains seven factors, with the eighth eigenvalue slightly below the threshold (0.968). In contrast, FE-EFA retains eight factors, as the eighth eigenvalue exceeds unity (1.096). The first seven eigenvalues are similar across the two methods, and the difference occurs at the retention boundary. This pattern is also observed in the scree plot (Figure 1).

#### 5.1.2. Marginal Factor Under Uncertainty-Aware Representation

The additional factor identified by FE-EFA originates from variation that is weakly represented under point-valued encoding. In Likert-scale data, responses often lie between adjacent categories, especially for perception-based indicators.

Point-valued encoding assigns each response to a single category, which may reduce sensitivity to subtle covariance patterns. The FE-EFA approach distributes responses across adjacent categories, allowing intermediate states to contribute to the correlation structure.

This difference becomes critical near the retention threshold. Variation that is insufficient to support an additional factor under point-valued encoding becomes detectable when ordinal ambiguity is preserved. The eighth factor can therefore be interpreted as a marginal dimension that is present but compressed in the original representation.

#### 5.1.3. Seven-Factor Structure Under Point-Valued EFA

The seven-factor solution obtained from the point-valued EFA provides a baseline representation of the latent structure. The factors correspond to Image and Cultural Display, Route Planning, Travel Comfort, Comprehensive Benefits, Service Facilities, Operation and Management, and Community Integration.

This structure is consistent with the conceptual design of the indicator system and captures the main dimensions of tramway cultural tourism evaluation. However, the rotated loading matrix indicates that some factor boundaries are not clearly separated. Several indicators show moderate secondary loadings, particularly those related to spatial organization, accessibility, and community interaction.

These patterns suggest that certain dimensions are partially aggregated under point-valued encoding. The seven-factor solution is therefore useful as a reference, but may not fully capture finer distinctions among related constructs. The detailed interpretation is reported in Table 5.

#### 5.1.4. Eight-Factor Structure Under FE-EFA

Under FE-EFA, an eight-factor structure is retained. The overall configuration is similar to the baseline solution, but with more differentiated patterns in specific domains.

A key difference is the decomposition of the original “Comprehensive Benefits” factor into two dimensions: Promotion Effectiveness and Financial Viability. Indicators related to media exposure and image dissemination are grouped into the former, while revenue-related indicators are assigned to the latter. This separation reflects a more explicit distinction between symbolic and economic effects.

Other factors remain consistent with the baseline structure, including image and cultural display, travel comfort, route planning, operational management, service facilities, and community integration. At the same time, indicator clustering within factors appears more conceptually consistent.

The additional factor mainly involves indicators located near the boundary between spatial planning, media perception, and community-related interaction. This suggests that FE-EFA separates relationships that are partially merged under point-valued encoding. The full interpretation is presented in Table 6.

#### 5.1.5. Comparison of the Two Structures

The two solutions are similar at the overall level, with consistent main dimensions across methods. Differences appear at a finer level.

FE-EFA retains an additional marginal factor and separates certain aggregated dimensions into more specific components. Indicator grouping is also more consistent within factors.

These results suggest that uncertainty-aware representation may reveal more differentiated factor-level organization in this empirical case. Rather than introducing new constructs, it makes existing relationships more distinguishable, particularly near the boundaries between related dimensions.

### 5.2. Parameter Sensitivity and Structural Robustness

To examine whether the FE-EFA results depend on a specific fuzziness parameterization, a sensitivity analysis was conducted across alternative values of the uncertainty ratio $r=\beta_{b}/\beta_{c}$. Because the membership function is normalized by the factor $\left(2\beta_{b}+\beta_{c}\right)$, the effective shape of the fuzzy representation depends primarily on r rather than on the absolute magnitudes of $\beta_{b}$ and $\beta_{c}$. The sensitivity analysis therefore focuses on changes in r as the principal uncertainty-control parameter.

Five representative parameter settings were examined, ranging from the crisp baseline (r=0) to higher levels of adjacent-category redistribution. The parameter setting adopted in the main FE-EFA corresponds to a moderate uncertainty level $\left(\beta_{b}=0.20,\;\beta_{c}=0.60,\;r\approx 0.333\right)$, which lies within the empirically stable region identified through sensitivity testing.

For each parameter setting, the fuzzy membership matrix, membership-space covariance matrix, entropy measures, Jensen–Shannon divergence, and factor extraction results were recalculated using the same analytical procedure. The corresponding sensitivity analysis results are summarized in Table 7.

The sensitivity analysis demonstrates that the FE-EFA framework remains structurally stable across a broad range of uncertainty ratios. The membership patterns corresponding to each uncertainty ratio were generated according to the normalized coefficients defined in Section 4.1. As the uncertainty ratio r increases, entropy and Jensen–Shannon divergence gradually increase, indicating stronger redistribution of ordinal membership across adjacent categories. At the same time, the eighth eigenvalue decreases progressively, reflecting a smoother covariance structure in membership space under higher fuzziness levels. Nevertheless, the eighth eigenvalue consistently remains above the Kaiser threshold (λ>1) throughout the tested parameter range, and the retained factor number remains stable at eight. These results indicate that the latent structure identified by the FE-EFA framework is robust to moderate variations in fuzziness parameterization rather than being driven by a single arbitrarily selected parameter setting.

### 5.3. Supplementary Robustness Comparisons

To further assess the robustness of the FE-EFA solution, supplementary analyses were conducted using point-valued Pearson EFA, polychoric-correlation EFA, bootstrap resampling, and split-sample replication. The key results of these supplementary robustness checks are summarized in Table 8.

The results show that both the point-valued Pearson EFA and the polychoric-correlation EFA retained seven factors under the Kaiser criterion, with eighth eigenvalues of 0.968 and 0.897, respectively. In contrast, FE-EFA retained eight factors, with an eighth eigenvalue of 1.096. This suggests that the additional factor identified by FE-EFA is not merely a consequence of using an ordinal correlation metric, but is associated with the uncertainty-aware fuzzy representation of ordinal responses.

The loading results also indicate more differentiated allocation patterns under FE-EFA. Under the naturally retained factor solutions determined by the Kaiser criterion, the Pearson EFA produced four cross-loading indicators and the polychoric EFA produced six, whereas FE-EFA produced only two using a secondary-loading threshold of 0.30. This result suggests that the uncertainty-aware fuzzy representation reduced ambiguous indicator allocation and produced fewer cross-loadings with more distinct factor allocation in this empirical case.

Bootstrap resampling further supported the stability of the FE-EFA solution. Across 300 bootstrap replications, the mean eighth eigenvalue of FE-EFA was 1.105, with a 95% interval of [1.040, 1.173]. By comparison, the mean eighth eigenvalue of the Pearson EFA was 0.974, with a 95% interval of [0.878, 1.067]. At least eight factors were retained in 99.7% of the FE-EFA bootstrap samples, compared with 27.7% of the Pearson EFA bootstrap samples.

Split-sample replication also indicated that the factor-loading patterns were reproducible. The sample was randomly divided into two subsamples, and matched factor structures were compared using Tucker’s coefficient of congruence. The FE-EFA solution showed consistently high congruence values across matched factors, ranging from 0.989 to 0.997. Comparable high congruence was also observed for the Pearson and polychoric solutions, indicating that the general factor pattern was not sample-specific. Taken together, these results support the empirical plausibility and stability of the FE-EFA solution, while confirming that the proposed method should be interpreted as an exploratory representation-level extension rather than a substitute for confirmatory validation.

### 5.4. Controlled Comparison Under a Unified Eight-Factor Specification

To distinguish the effect of factor retention from structural organization, both analytical routes were re-estimated under a fixed eight-factor specification. This controlled setting allows a direct comparison of indicator allocation and factor clarity when dimensionality is held constant.

#### 5.4.1. Overall Structural Correspondence

At the overall level, the two methods produce comparable factor frameworks. The main thematic dimensions remain identifiable in both solutions, indicating that the underlying conceptual structure of the indicator system is stable across analytical routes.

Differences become apparent when examining the internal organization of factors. With the number of factors fixed, variations emerge in indicator allocation and the distinctness of factor boundaries. The FE-EFA solution shows more conceptually concentrated grouping of related indicators, whereas the point-valued solution exhibits a relatively more diffuse structure.

#### 5.4.2. Indicator Allocation and Cross-Loading Patterns

The clearest difference between the two methods is observed in cross-loading patterns. Under the unified eight-factor specification imposed for controlled comparison, the point-valued solution contains a larger number of moderate cross-loadings, while the FE-EFA solution shows a more distinct allocation of indicators.

Using a secondary-loading threshold of 0.30 under the forced eight-factor specification, five cross-loading indicators are identified in the point-valued solution, compared to two in the FE-EFA solution. This reduction indicates a more distinct allocation of indicators across latent constructs under uncertainty-aware preprocessing.

Table 9 summarizes the comparison between the two solutions. While the cumulative variance explained by the point-valued solution is higher, the FE-EFA solution shows fewer cross-loadings and more conceptually concentrated indicator clustering.

The difference is particularly evident for indicators related to spatial planning, accessibility, media perception, and community interaction. In these domains, responses are more likely to involve ambiguity between adjacent ordinal categories. The FE-EFA representation appears to reduce overlap between factors in such cases.

#### 5.4.3. Structural Interpretation Under FE-EFA

The reduction in cross-loadings is associated with more distinct factor boundaries and more consistent interpretation. In the FE-EFA solution, indicators are more consistently grouped within factors, and the distinction between related dimensions becomes more explicit.

By contrast, the point-valued solution shows broader and partially overlapping factors, especially in domains where perception-based evaluation is involved. This pattern is consistent with the aggregation effect observed under discrete encoding.

It should be noted that the difference under FE-EFA is not reflected in all statistical indicators. Under the unified specification, the cumulative explained variance remains higher in the point-valued solution (65.995%) than in the FE-EFA solution (58.947%).

**Figure 2:**
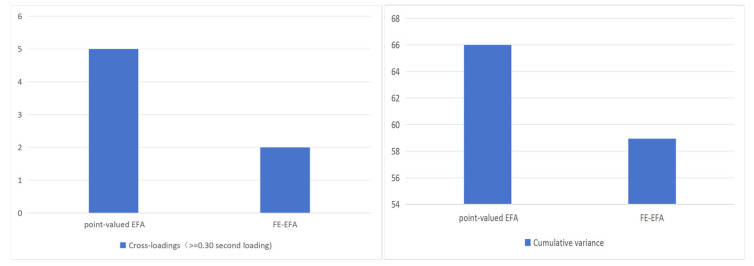
Loading distinctiveness comparison under the unified eight-factor specification.

However, the main contribution of FE-EFA in this study lies in its suggested more differentiated allocation of indicators across latent constructs rather than variance maximization. As illustrated in Figure 2, the FE-EFA solution provides a more differentiated structural representation when ordinal uncertainty is taken into account.

### 5.5. Entropy, Jensen–Shannon Divergence, and Weight Reallocation

To further examine how uncertainty-aware preprocessing affects the internal representation of indicators, this section analyzes four aspects: entropy-based dispersion, indicator-level Jensen–Shannon (JS) divergence, entropy-adjusted weight reallocation, and structural divergence in correlation patterns. These measures provide a complementary information-theoretic perspective beyond factor loading patterns.

#### 5.5.1. Entropy as a Measure of Representation Dispersion

Entropy was used to quantify the dispersion of indicator evaluations under the point-valued and FE-EFA representations. In this context, entropy reflects the degree of uncertainty in the distribution of responses across ordinal categories.

**Figure 3:**
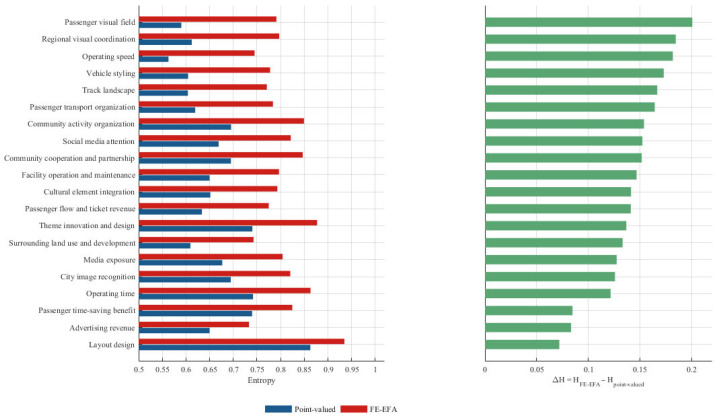
Entropy comparison (blue: point-valued; red: FE-EFA) and entropy increase (green: $\Delta H=H_{FE-EFA}-H_{Point-valued}$) f or the top 20 indicators ranked by representation-induced entropy change.

As shown in Figure 3, entropy values under FE-EFA are consistently higher than those under the point-valued representation across all indicators, with an average entropy of 0.8688, indicating an overall increase in distributional dispersion. However, the magnitude of entropy increase is not uniform across indicators. Indicators located near the middle range of the Likert scale exhibit larger entropy gains, suggesting greater adjacent-category ambiguity in perception-based evaluations. By contrast, indicators with more polarized response distributions, particularly those concentrated near boundary categories, display smaller entropy changes due to the boundary-folding mechanism embedded in the fuzzy transformation.

This pattern is consistent with the behavioral interpretation of the fuzzy representation mechanism. Responses near the center of the ordinal scale are more likely to reflect hesitation, transitional judgments, or overlapping perceptions, resulting in broader membership distributions across adjacent categories. In contrast, extreme responses remain comparatively concentrated after transformation because fewer neighboring categories are available for redistribution.

The observed entropy increase is therefore not attributable to additional statistical noise, but rather to the recovery of ordinal ambiguity that is suppressed under point-valued encoding. Unlike conventional encoding, which assigns each observation exclusively to a single category, FE-EFA permits partial membership across adjacent categories, thereby expanding the support of the response distribution and reducing artificial concentration effects.

From an information-theoretic perspective, these results suggest that FE-EFA preserves representation-level variability associated with adjacent-category ambiguity embedded in subjective ordinal evaluations. This additional variability may provide a representation that is more consistent with transitional ordinal judgments for subsequent structural weighting and multidimensional assessment.

#### 5.5.2. Indicator-Level Distributional Divergence

To assess how the fuzzy representation modifies the distribution of ordinal responses, Jensen–Shannon (JS) divergence was computed for each indicator by comparing the point-valued distribution with the corresponding fuzzy distribution.

Unlike entropy, which measures dispersion within a single representation, JS divergence captures the redistribution between representations. It therefore quantifies the extent to which the FE-EFA transformation departs from the original point-valued encoding.

Table 10 reports the 10 indicators with the highest JS divergence values. The largest divergence is observed for Passenger visual field (0.0302), followed by Regional visual coordination (0.0265) and Operating speed (0.0262). At the same time, the average JS divergence across all indicators remains relatively small (0.0133). This interpretation is consistent with domain-specific applications in engineering and signal analysis, where very small JS divergence values (e.g., around 0.05) are treated as indicating negligible differences under specific task settings [29]. However, such thresholds are application-dependent and not universal.

Indicators with higher JS divergence are mainly concentrated in perceptual and interaction-related domains, where responses tend to span adjacent categories. In these cases, the fuzzy transformation leads to more pronounced redistribution across neighboring categories, making localized distributional variation more visible. Conversely, indicators with low JS divergence remain largely stable under both representations, indicating well-defined response distributions with limited ambiguity. Overall, JS divergence in this study should be interpreted as a diagnostic measure of localized redistribution rather than as evidence of distortion or improvement.

#### 5.5.3. Weight Reallocation Under Entropy Constraints

**Figure 4:**
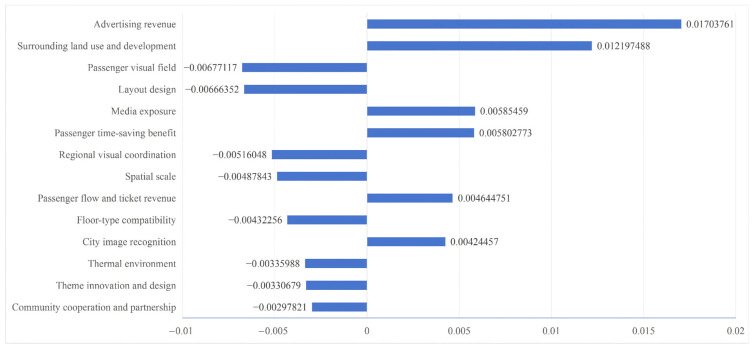
Top 15 entropy-adjusted weight adjustments under FE-EFA relative to point-valued encoding.

Based on the entropy results, indicator weights were recalculated by combining structural contribution with entropy-based distributional concentration. Figure 4 presents the top 15 weight adjustments under FE-EFA relative to the point-valued representation.

Overall, the two weighting schemes remain broadly comparable, indicating that the uncertainty-aware transformation does not fundamentally alter the overall importance structure of the indicator system. However, localized deviations are observed at the indicator level, reflected as both positive and negative adjustments rather than uniform shifts.

As shown in Figure 4, Advertising revenue and Surrounding land use and development exhibit the largest positive adjustments, whereas Passenger visual field and Layout design show the most evident decreases. This pattern suggests that FE-EFA selectively reallocates indicator importance according to the informational characteristics of ordinal response distributions.

More specifically, indicators with relatively concentrated fuzzy distributions and stable structural contributions tend to receive higher entropy-adjusted weights. In contrast, indicators associated with stronger distributional dispersion or greater adjacent-category ambiguity may experience relative down-weighting. Therefore, entropy functions not only as a descriptive measure of uncertainty, but also as a regularizing component that links representation-level dispersion to final weighting outcomes.

#### 5.5.4. Structural Divergence in Correlation Patterns

In addition to distributional changes, FE-EFA modifies the correlation structure among indicators. To examine this effect, absolute differences between the point-valued and fuzzy correlation matrices were computed.

Table 11 reports the indicator pairs with the largest changes in pairwise correlations. The most pronounced differences involve indicators related to spatial design, media perception, and operational performance. These domains are typically associated with perceptual and context-dependent evaluations.

A consistent directional pattern is observed. Correlations that are weakly negative under point-valued encoding often become close to zero or weakly positive under FE-EFA. This suggests that some negative associations may be influenced by rigid category boundaries rather than stable underlying relationships.

At the aggregate level, structural changes remain moderate. The average absolute correlation difference is 0.0569, with a maximum of 0.2015, indicating that most pairwise relationships are only moderately affected by the uncertainty-aware transformation. Although several localized changes are observed among specific indicator pairs, the overall correlation pattern remains broadly consistent between the point-valued and FE-EFA representations. This suggests that the proposed transformation refines selected dependency relationships without substantially altering the global structure of inter-indicator associations.

These changes can be interpreted as a redistribution effect across adjacent ordinal categories. By allowing partial membership across adjacent categories, FE-EFA reduces abrupt transitions and produces more continuous covariance relationships.

#### 5.5.5. Integrated Interpretation

The combined results from entropy, JS divergence, weight adjustment, and correlation analysis show that FE-EFA modifies the representation of ordinal data in a controlled and localized way without altering its overall structure.

Entropy increases due to the redistribution of category membership, while JS divergence indicates that these changes remain controlled and localized. Weight reallocation reflects adjustments in indicator importance based on both structural relevance and distributional concentration. At the same time, correlation patterns exhibit moderate but systematic changes.

Overall, these results suggest that uncertainty-aware preprocessing may make certain representation-level differences in the factor model more visible and interpretable in this empirical case, particularly for perception-based indicators where responses are inherently ordinal and transitional.

### 5.6. Practical Implications and Summary of Findings

The comparative results in Section 5.1, Section 5.2, Section 5.3 and Section 5.4 show that introducing uncertainty-aware representation leads to consistent differences in factor structure, indicator allocation, and representation-level information distribution.

Under the same retention criterion, FE-EFA identifies an additional marginal dimension that remains close to the retention boundary in the point-valued solution. When the number of factors is fixed, the FE-EFA results exhibit more distinct loading patterns and fewer cross-loadings, suggesting a more differentiated allocation of latent constructs in this dataset. These differences are further reflected at the distributional level, where entropy and Jensen–Shannon divergence reveal systematic shifts in how ordinal responses are represented and aggregated.

At the indicator level, entropy-based weighting produces directional adjustments rather than uniform changes, with some indicators gaining importance while others decrease. This suggests that uncertainty-aware preprocessing does not simply rescale the original results, but modifies the internal balance of the system by incorporating both structural contribution and distributional characteristics.

From a practical perspective, these findings imply that perception-based evaluation results may depend not only on the observed responses but also on how ordinal information is represented prior to analysis. In applied settings such as cultural tourism or transport service evaluation, this has implications for how key dimensions are identified and how indicator importance is interpreted in decision-making contexts.

### 5.7. Limitations and Future Research

The present study examines how uncertainty-aware response representation influences exploratory factor structures under a controlled comparison with conventional point-valued encoding. While supplementary robustness checks were conducted using polychoric-correlation EFA, bootstrap resampling, split-sample replication, and simple-structure residual validation, the current validation remains exploratory. These checks provide supporting evidence for the empirical stability of the FE-EFA solution, but they do not constitute a full confirmatory validation framework. In particular, formal SEM-based CFA, item response theory models, and external validation samples were not incorporated in the present study. Future research should further assess the robustness, structural consistency, and generalizability of the proposed framework using independent datasets and confirmatory modeling approaches.

## 6. Conclusions

This study proposes a parameterized fuzzy–entropy exploratory factor analysis (FE-EFA) framework for the analysis of ordinal Likert-scale perception data. By introducing a fuzzy membership representation and integrating entropy-based measures, the framework extends conventional EFA at the data representation stage while preserving its core analytical structure.

The results suggest that incorporating uncertainty into ordinal data representation can affect both factor retention and structural organization. Under a unified empirical setting, FE-EFA identifies an additional marginal factor that remains near the retention boundary in the point-valued solution. When dimensionality is controlled, the FE-EFA results suggest more distinct factor allocation, fewer cross-loadings, and more conceptually concentrated indicator clustering in this empirical case.

From an information-theoretic perspective, the proposed framework makes representation-level distributional characteristics explicit that are suppressed under point-valued encoding. The increase in entropy reflects the preservation of ordinal ambiguity, while the relatively low Jensen–Shannon divergence indicates that the overall distributional structure remains stable. In addition, entropy-adjusted weighting reveals systematic shifts in indicator importance, linking structural contribution with distributional concentration.

These findings suggest that the representation of ordinal data plays a substantive role in factor-analytic outcomes. Rather than altering the conceptual structure, the FE-EFA framework provides an exploratory representation-level perspective for examining latent relationships by making implicit uncertainty analytically visible. This may provide an additional perspective for interpreting perception-based evaluation results.

In practical terms, the proposed approach offers a flexible exploratory tool for analyzing perception data in cultural tourism, transport evaluation, and related domains. It allows uncertainty to be incorporated without modifying survey design, making it suitable for a wide range of existing datasets. From a management perspective, the separation between Promotion Effectiveness and Financial Viability provides a more differentiated basis for tramway cultural tourism development. Promotion-related indicators, such as city image recognition, media exposure, and social media attention, should be managed through targeted communication strategies, visual identity building, and cultural-brand dissemination. In contrast, financial-viability indicators, including passenger flow and ticket revenue, advertising revenue, time-saving benefits, and surrounding land-use development, require operational and revenue-oriented strategies. Treating these two dimensions separately may help avoid conflating symbolic visibility with economic performance and support more targeted policy and management interventions.

The present study focuses on a specific parameterization of uncertainty and a single empirical context. Future research may examine alternative forms of uncertainty representation, explore applications in different domains, and further investigate the interaction between representation, information measures, and latent structure identification.

## Figures and Tables

**Table 1:** Demographic characteristics of respondents.

Category	Group	Count	Percentage
Residency	Local/long-term	930	74.88%
	Non-local	312	25.12%
Age	18–24	595	47.91%
	25–34	397	31.96%
	35–44	162	13.04%
	45 and above	88	7.09%
Gender	Male	571	45.97%
	Female	646	52.01%
	Prefer not to say	25	2.01%
Education	Undergraduate student	522	42.03%
	Graduate student	348	28.02%
	Non-student	372	29.95%

**Table 2:** Data suitability and factor-retention summary.

Item	Value	Interpretation
Sample size	1242	Adequate for stable factor estimation relative to 32 indicators
KMO	0.8755	Indicates strong sampling adequacy for factor analysis
Bartlett’s test	$\chi^{2}$ = 25,147.27, df = 496, *p* < 0.001	Rejects identity matrix; correlations are sufficient for structure extraction
Factor retention by Kaiser criterion (point-valued data)	7	Provides baseline dimensionality under conventional point-valued representation for subsequent comparative analysis

**Table 3:** Fuzzy membership vectors under the selected parameterization (r≈0.333).

Response $x_{ij}$	$\mu_{1}$	$\mu_{2}$	$\mu_{3}$	$\mu_{4}$	$\mu_{5}$	Type
1	0.80	0.20	0	0	0	Boundary (folded)
2	0.20	0.60	0.20	0	0	Interior (symmetric)
3	0	0.20	0.60	0.20	0	Interior (symmetric)
4	0	0	0.20	0.60	0.20	Interior (symmetric)
5	0	0	0	0.20	0.80	Boundary (folded)

**Table 4:** Eigenvalue comparison and factor retention based on the Kaiser criterion.

Component	Point-Valued EFA Eigenvalue	>1	FE-EFA Eigenvalue	>1
1	6.884	yes	6.448	yes
2	5.188	yes	3.988	yes
3	3.011	yes	2.994	yes
4	2.855	yes	2.654	yes
5	1.863	yes	1.898	yes
6	1.605	yes	1.557	yes
7	1.346	yes	1.359	yes
8	0.968	no	1.096	yes

**Table 5:** Interpretation of the seven-factor structure under point-valued EFA.

Factor	Factor Name	Main Indicators	Interpretation
Factor 1	Image and Cultural Display	Track landscape; Vehicle styling; Regional visual coordination; Passenger visual field; Cultural element integration	Represents the visual image and cultural expression of the tramway system, emphasizing landscape quality, vehicle appearance, spatial visual experience, and the integration of cultural elements into the tourism environment.
Factor 2	Route Planning	Number of scenic spots covered; Integration with bus system; Station spacing rationality; Matching degree with tourism flow	Represents the rationality and connectivity of route design, including attraction coverage, multimodal integration, stop spacing, and alignment with tourism movement patterns.
Factor 3	Travel Comfort	Thermal environment; Spatial scale; Lighting environment; Floor-type compatibility; Layout design	Represents the environmental and spatial comfort experienced by passengers during travel, including thermal conditions, interior scale, lighting, floor compatibility, and layout quality.
Factor 4	Comprehensive Benefits	Surrounding land use and development; Passenger flow and ticket revenue; Advertising revenue; Passenger time-saving benefit; City image recognition; Media exposure; Social media attention	Represents the broader comprehensive benefits generated by the tramway system, including economic returns, efficiency gains, land-use effects, and wider image and publicity impacts.
Factor 5	Service Facilities	Tourist service facilities; Accessibility facilities; Tourism information service; Technological innovation and intelligence	Represents the quality and completeness of supporting facilities and service functions, including tourism-oriented services, accessibility support, information provision, and intelligent technology applications.
Factor 6	Operation and Management	Facility operation and maintenance; Operating time; Passenger transport organization; Operating speed	Represents the operational efficiency and management quality of the tramway system, covering maintenance, service hours, passenger organization, and operating performance.
Factor 7	Community Integration	Theme innovation and design; Community activity organization; Community cooperation and partnership	Represents the extent to which the tramway system is integrated with local community life through thematic design, activity participation, and cooperative relationships with community stakeholders.

**Table 6:** Interpretation of the eight-factor structure under FE-EFA.

Factor	Factor Name	Main Indicators	Interpretation
F1	Image and Cultural Display	Track landscape; Vehicle styling; Regional visual coordination; Passenger visual field; Cultural element integration	Represents the aesthetic aspects of the tourism transit system, emphasizing cultural representation and visual harmony.
F2	Travel Comfort	Thermal environment; Spatial scale; Lighting environment; Floor-type compatibility; Layout design	Represents the physical comfort experienced by passengers, including factors like climate control, spatial design, and lighting.
F3	Promotion Effectiveness	City image recognition; Media exposure; Social media attention	Represents the symbolic visibility and promotional impact of the tramway system, including city image recognition, media exposure, and social media attention.
F4	Route Planning	Number of scenic spots covered; Integration with bus system; Station spacing rationality; Matching degree with tourism flow	Represents the design and planning of the routes, including coverage of tourist spots, connectivity with other transport modes, and rationality of station placement.
F5	Operation and Management	Facility operation and maintenance; Operating time; Passenger transport organization; Operating speed	Represents the efficiency and management of the transit system, including operational timelines, maintenance, and passenger handling.
F6	Service Facilities	Tourist service facilities; Accessibility facilities; Tourism information service; Technological innovation and intelligence	Represents the infrastructure supporting tourists, such as accessibility, technology use, and service availability.
F7	Community Integration	Theme innovation and design; Community activity organization; Community cooperation and partnership	Represents the interaction between the transit system and local communities, focusing on community engagement and cooperative initiatives.
F8	Financial Viability	Surrounding land use and development; Passenger flow and ticket revenue; Advertising revenue; Passenger time-saving benefit	Represents the financial and operational sustainability of the transit system, including the generation of revenue through passenger flow, advertising, and land development.

**Table 7:** Sensitivity analysis of the FE-EFA uncertainty ratio.

$r\;=\;\beta_{b}/\beta_{c}$	8th Eigenvalue	Retained Factors	Mean Entropy	Mean JS Divergence
0.000	0.968	7	0.767	0.000
0.100	1.139	8	0.816	0.002
0.200	1.125	8	0.843	0.007
0.300	1.109	8	0.861	0.011
0.333	1.096	8	0.869	0.013
0.400	1.092	8	0.873	0.015
0.500	1.079	8	0.881	0.018

**Table 8:** Robustness comparison across Pearson EFA, polychoric EFA, and FE-EFA.

Metric	Pearson EFA	Polychoric EFA	FE-EFA
Correlation type	Pearson correlation	Polychoric correlation	Fuzzy membership-space correlation
Retained factors (Kaiser criterion)	7	7	8
8th eigenvalue	0.968	0.897	1.096
Cross-loading indicators (≥0.30)	4	6	2
Bootstrap replications	300	–	300
Mean 8th eigenvalue (bootstrap)	0.974	–	1.105
95% CI of 8th eigenvalue	[0.878, 1.067]	–	[1.040, 1.173]
Samples retaining ≥ 8 factors	27.7%	–	99.7%
Split-sample congruence (Tucker’s coefficient)	High	High	0.989–0.997
Loading distinctiveness	Moderate	Moderate	Relatively high
Indicator clustering	More dispersed	More dispersed	More conceptually concentrated

**Table 9:** Unified 8-factor comparison summary.

Metric	Point-Valued EFA	FE-EFA
Retained factors (Kaiser)	7	8
Cumulative variance (8 factors)	65.995%	58.947%
Number of cross-loadings (≥0.30)	5	2
Loading distinctiveness	Moderate	Relatively high
Indicator clustering	More dispersed	More conceptually concentrated

**Table 10:** Top 10 indicators ranked by Jensen–Shannon divergence.

Rank	Indicator	JS Divergence
1	Passenger visual field	0.0302
2	Regional visual coordination	0.0265
3	Operating speed	0.0262
4	Vehicle styling	0.0244
5	Track landscape	0.0236
6	Passenger transport organization	0.0222
7	Social media attention	0.0201
8	Community activity organization	0.0195
9	Community cooperation and partnership	0.0195
10	Passenger flow and ticket revenue	0.0190

**Table 11:** Top 10 structural changes in pairwise correlations.

Indicator Pair		Point-Valued Correlation	FE-EFA Correlation	|Δρ|
Layout design	Media exposure	−0.1660	0.0354	0.2015
Layout design	City image recognition	−0.1348	0.0488	0.1836
Spatial scale	Media exposure	−0.1325	0.0451	0.1776
Floor-type compatibility	Media exposure	−0.1326	0.0355	0.1681
Lighting environment	Media exposure	−0.1360	0.0220	0.1580
Station spacing rationality	Facility operation and maintenance	−0.1419	0.0154	0.1574
Layout design	Operating speed	−0.1359	0.0192	0.1551
Integration with bus system	Facility operation and maintenance	−0.1541	0.0008	0.1550
Spatial scale	City image recognition	−0.1177	0.0370	0.1546
Floor-type compatibility	Social media attention	−0.1350	0.0166	0.1516

## Data Availability

The data and supplementary statistical outputs supporting the findings of this study are available from the corresponding author upon reasonable request, subject to anonymization and institutional data-sharing requirements.
